# Survival and Functional Immune Reconstitution After Haploidentical Stem Cell Transplantation in *Atm*-Deficient Mice

**DOI:** 10.3389/fimmu.2021.693897

**Published:** 2021-06-29

**Authors:** Ruth Pia Duecker, Lucia Gronau, Patrick C. Baer, Stefan Zielen, Ralf Schubert

**Affiliations:** ^1^ Division for Allergy, Pneumology and Cystic Fibrosis, Department for Children and Adolescence, Goethe-University, Frankfurt am Main, Germany; ^2^ Division of Nephrology, Department of Internal Medicine III, Goethe-University, Frankfurt am Main, Germany

**Keywords:** Ataxia-telangiectasia, HSCT, haploidentical, ATM, GvHD, T-lymphocytes

## Abstract

Hematopoietic stem cell transplantation (HSCT) has been proposed as a promising therapeutic opportunity to improve immunity and prevent hematologic malignancies in Ataxia-telangiectasia (A-T). However, experience in the transplantation strategy for A-T patients is still scarce. The aim of this study was to investigate whether different approaches of HSCT are feasible in regard to graft versus host response and sufficient concerning functional immune reconstitution. *Atm*-deficient mice were treated with a clinically relevant non-myeloablative host-conditioning regimen and transplanted with CD90.2-depleted, green fluorescent protein (GFP)-expressing, and ataxia telangiectasia mutated (ATM)-competent bone marrow donor cells in a syngeneic, haploidentical or allogeneic setting. Like syngeneic HSCT, haploidentical HSCT, but not allogeneic HSCT extended the lifespan of *Atm*-deficient mice through the reduction of thymic tumors and normalized T-cell numbers. Donor-derived splenocytes isolated from transplanted *Atm*-deficient mice filled the gap of cell loss in the naïve T-cell population and raised CD4 cell functionality up to wild-type level. Interestingly, HSCT using heterozygous donor cells let to a significantly improved survival of *Atm*-deficient mice and increased CD4 cell numbers as well as CD4 cell functionality equivalent to HSCT using with wild-type donor cells. Our data provided evidence that haploidentical HSCT could be a feasible strategy for A-T, possibly even if the donor is heterozygous for ATM. However, this basic research cannot substitute any research in humans.

## Introduction

Ataxia-telangiectasia (A-T) is a genomic instability syndrome with progressive neurological deterioration, oculocutaneous telangiectasia, high risk for malignancy, recurrent pulmonary infections, and variable immunodeficiency (1). Immunodeficiency affects numbers as well as functionality of the B and T cell repertoire (2, 3). A-T is based on mutations in the ataxia telangiectasia-mutated (*ATM*) gene, which is located on chromosome 11q22.3 and regulates complex signaling cascades including DNA damage recognition and repair responses (4–6). Besides lung failure, an elevated cancer susceptibility due to genomic instability and impaired DNA damage responses accounts for morbidity and mortality in A-T (7–10). Occurrence of chromosomal aberrations in T and B lymphocytes are responsible for lymphoid tumors in approximately 10-15% of the patients, making A-T to the disease with the highest malignancy in childhood (11).

Up to date no curative therapy for A-T exist (12). One encouraging approach represents hematopoietic stem cell transplantation (HSCT) to ameliorate the immunodeficiency and prevent the development of leukemia and lymphomas. In *Atm*-deficient mice, which typically die at the age of 3−6 months, the inhibition of tumorigenesis has been impressively demonstrated (13). Moreover, it has been shown that HSCT restores T-cell (sub)populations in *Atm*-deficient mice as well as in patients with Ataxia-telangiectasia (14, 15). However, experience in transplantation of A-T patients is scarce and, therefore, using the right conditioning regime and choosing the right donor are of utmost importance. A potential donor such as a HLA-identical sibling is only applicable for a minority of the patients (16). For patients who need a stem cell transplant but do not have an HLA-matched related or unrelated donor, recent medical advances have made the use of a partially matched or haploidentical related donor possible. Thus, haploidentical HSCT increases the chance of finding a donor since everyone has at least one haploidentical relative such as a parent, sibling, or child (17).

The present study aimed to compare different transplantation settings including haploidentical HCST regarding inhibition of tumor development, GvHD and functional immune reestablishment in *Atm*-deficient mice. In addition, the study also focused on donor derived ATM heterogeneity in regard of haploidentical donors such as biological parents or siblings.

## Material and Methods

### Animals

In this proof of principle experiments, we used a total of 68 *Atm*-deficient, 56 wildtype mice and 9 heterozygote mice. For HSCT experiments mice were randomly assigned to the treatment groups and mice where HSCT failed were excluded from the experiments.

The animal studies were performed according to the protocols approved by the German Animal Subjects Committee (Gen. Nr. FK/1034). *Atm*-deficient mice (*Atm* ^tm1Awb^; 8 to 10 weeks old), in a 129SvEv background, were housed in IVC cages on a standard light and dark cycle of 12 h with access to food and water *ad libitum* until harvest. Mice were checked 3 times per week and sacrificed when clinical signs like gradual weight loss indicating emerging thymic lymphomas. Weight was taken twice a week during the observation period.

One hundred percent of our untreated *Atm*-deficient mice die at the age of 3-6 months due to the development of massive thymic lymphoblastic lymphomas. These tumors grow rapidly causing death from compression of the heart and lungs. As a result, these mice experience significant weight loss (>20%) and signs of disturbed general condition due to an insufficient food and water intake in a very short time and then mice are euthanized to avoid any further animal suffering.

For the HSCT experiments, 11 recipient mice and 9 donor mice (129S6/SvEvGFP^+^) for the syngeneic transplantation setting, 12 recipient mice and 8 donor mice (F1 generation of C57BL/6J-129S6/SvEv GFP^+^) for haplo-HSCT, 13 recipients and 8 donor mice (C57BL/6J GFP^+^) for allo-HSCT, and 7 *Atm*-deficient mice and 4 heterozygous donor mice for the het-HSCT setting were used.

### Mixed Lymphocyte Reaction

For the mixed lymphocyte reaction (MLR), dendritic cells (DC) isolated from bone marrow were obtained from *Atm* ^+/+^ and *Atm* ^-/-^ mice and were pretreated with Mitomycin C (MMC, 50 µg/mL, Sigma-Aldrich, Steinheim, Germany), preventing the cells to replicate. Spleen cells from syngeneic, haploidentical and allogeneic donor mice were used as responder cells. For the syngeneic stimulation, spleen cells from GFP^+^ ATM-competent donor mice were used. As haploidentical donor cells spleen cells of the F1-generation of mated 129S6/SvEv-Atm^(tm1)Awb/F^ and C57BL/6^Tg(ACTb-EGFP)10sb/J^ mice were used. Allogeneic splenocytes were taken from RjOrl : Swiss mice (Janvier Labs). The isolation and culture of DCs from progenitor cells of the bone marrow of *Atm*-deficient and wild-type mice was performed slightly modified accordingly to Lutz et al. (18). Additional detail on the method is provided in an online data supplement. Briefly, femur and tibiae from 6 – 8 weeks old wild-type and Atm-deficient mice were removed and purified from the surrounding muscle tissue by rubbing carefully with kim wipes. Thereafter, intact bones were left in 70% ethanol for 2 - 5 min for disinfection and washed with RPMI1640 (Gibco, Carlsbad, USA). Bones were cut open with scissors and the marrow flushed with culture medium [RPMI1640, 100 U/mL penicillin-streptomycin, 2 mM L-Glutamine, 10% heat-inactivated FBS (Sigma, Steinheim, Germany)] using a syringe with a 0.5 mm diameter needle (25 G). The BM cells were separated by pipetting and passed through a 70 μm cell strainer. The cell strainer was rinsed with PBS three times and bone marrow cells were centrifuged (600 x g, 10 minutes, at room temperature). Subsequently, the cells were taken up in 10 mL culture medium containing 20 ng/mL recombinant mouse granulocyte macrophage-colony stimulating factor (rmGM-CSF, PeproTech, Hamburg, Germany), and cell density was adjusted to 2 x 106 per 9.5 cm^2^ dish and cultured at 37°C and 5% CO_2_ in the incubator. After 10 days of culture, the dendritic cells were harvested, labeled with 25 µmol carboxyfluoresceinsuccimidylester (CFSE, Thermo Fisher Scientific, Langenselbold, Germany) and tracked for successful proliferative precursors, according to manufacturer’s protocol, using the FACSVerse flow cytometer (Becton Dickinson, San Jose, CA, USA). The data were evaluated using FACSSuite software.

### HSCT in *Atm*-Deficient Mice

*Atm*-deficient mice received 0.125 mg/mL anti-CD4 (clone GK1.5) and 0.125 mg/mL anti-CD8 (clone 53 – 6.7) monoclonal antibody (Sigma, Steinheim, Germany) 7 days before bone marrow transplantation (BMT) and a second dose of each antibody in combination with cyclophosphamide (CP, 80 ng/mL, Endoxan, Baxter, Unterschleißheim, Germany) one day before BMT as non-myeloablative conditioning (**Figure 2A**). Bone marrow donor cells (BMDC) from 129S6/SvEv GFP-transfected wild-type mice (syngeneic) or from mice of the F1 generation of 129S6/SvEv wild-type mice and C57BL/6 mice (haploidentical), or C57BL/6 mice (allogeneic) were depleted using CD90.2 MicroBeads (CD90.2 MicroBeads, Miltenyi Biotec, Bergisch-Gladbach, Germany) according to manufacturer’s protocol to minimize GvH reactions. After conditioning 5 x 10^6^ CD90.2^-^ bone marrow donor cells derived from 129S6/SvEv GFP-transfected wild-type mice were injected intravenously into recipient mice (19, 20).

### GvHD Scoring

Some recipient mice began to show clinical signs of GvHD between 4 to 10 days after HSCT. The GvHD scoring system, developed by Cooke et al. were modified and used to quantitate disease progression. Following parameters were scored: fur texture, weight loss, skin integrity, activity, and posture (0 if absent or 1 if present) (21). Mice were scored twice per week and euthanized when weight loss was >20% or at any sign of disturbed general condition.

### Bone Marrow T-Cell Depletion

Bone marrow (BM) cells were collected by flushing femurs and tibias from 129S6/SvEv GFP-transfected wild-type mice (syngeneic) or from mice of the F1 generation of 129S6/SvEv wild-type mice and C57BL/6 mice (haploidentical), or C57BL/6 mice (allogeneic) donor mice into MACS buffer (1× PBS, 2 mM EDTA, 0.5% bovine serum albumin). After red blood cell lysis, BM cells were incubated with CD90.2 microbeads for 15 minutes on ice, washed once, and then placed on LS columns (Miltenyi Biotech GmbH, Germany) and magnetically separated using a MiniMACS (Miltenyi Biotech GmbH, Germany). The remaining CD90.2-positive cell fraction was routinely less than 5% of BM cells. Cells were washed and then resuspended in injection medium (DMEM (Gibco, Carlsbad, USA), 10% fetal bovine serum (FBS, Sigma-Aldrich, Steinheim, Germany), 100 U/mL penicillin-streptomycin (Gibco, Carlsbad, USA) prior to transplantation.

### Tracking the Migration of GFP^+^ Donor Cells in Recipient *Atm*-Deficient Mice After SCT

Two hundred µL of blood was collected by tapping the facial vein of the mice 6, 12 and 24 weeks after HSCT. EDTA containing whole blood samples were first blocked with anti-CD16/32 antibody (Becton Dickinson, Heidelberg, Germany) and then surface stained for anti-CD3-BV421, anti-CD4-PerCp and CD8-PE-Cy7 (BD, Heidelberg, Germany). After staining, erythrocytes were lyzed with FACS lysing solution (BD, Heidelberg, Germany). For analyzing, 10.000 events were acquired using a FACSVerse flow cytometer (BD, Heidelberg, Germany). The data were analyzed using the FACSuite software.

### T-cell Activation and Signaling *In Vitro*

The expression of CD69 following T-cell activation *via* TCR/CD28 stimulation was evaluated in splenocytes isolated from *Atm*-deficient mice 12 weeks after syngeneic HSCT and compared to cells from untreated *Atm*-deficient mice and wild-type mice. Further, the p-Erk intracellular signaling pathway after T-cells activation was investigated *in vitro* using the BD™ Phosflow Protocol for mouse splenocytes. Additional detail on the method is provided in an online data supplement. Briefly, the splenocytes were stimulated with either purified NA/LE Hamster anti-mouse CD3ϵ ([1 µg/mL], Clone 145-2C11, BD, Heidelberg, Germany) and purified NA/LE Hamster anti-mouse CD28 ([1 µg/mL], Clone 37.51, BD, Heidelberg, Germany) antibody or Concanavalin A (ConA [5 µg/mL], Merck, Darmstadt, Germany). The cells were incubated for 4 h at 37°C. Afterwards, the cells were blocked with CD16/32 (Biolegend, Koblenz, Germany) for 10 min and then surface stained using αCD3-V450 (Clone 17A2, BD, Heidelberg, Germany), αCD4-PerCP-Cy5.5 (Clone RM4-5, BD, Heidelberg, Germany), αCD8-PE (Clone 53-6.7, BD, Heidelberg, Germany), αCD19-APC (Clone 1D3, BD, Heidelberg, Germany), and αCD69-Alexa Fluor 647 (Clone H1.2F3, Biolegend, Koblenz, Germany) for 20 min in the dark. After washing the cells with PBS, they were resuspended in 300 µL PBS and 10,000 events were acquired by flow cytometry.

For intracellular detection of phosphorylated proteins, splenocytes were stimulated in a 37°C water bath *via* TCR/CD28 for 5 min with αCD3 and αCD28 antibodies. As a positive control served PBu2/Ionomycin which incubated for 15 min. The reaction was stopped by adding cytofix buffer (BD, Heidelberg, Germany) for 10 min at 37°C. After centrifugation, cells were washed with stain buffer (PBS + 2% FBS). Then 1 mL Perm III buffer (BD, Heidelberg, Germany) was added to the cells for 30 min on ice. After 2 washing steps, the cells were resuspended in stain buffer and blocked for 10 min with purified CD16/CD32 (Biolegend, Koblenz, Germany). Then the cells were stained with αCD3-V450, αCD4-PerCP-Cy5.5, and p-Erk-Alexa Fluor 647 (Clone 6B8B69, Biolegend, Koblenz, Germany) for 1 h and measured using a FACSVerse. The data were evaluated using the FACSuite software.

### Statistics

Statistical analyses were performed using GraphPad Prism 5.0 (GraphPad Software, San Diego, CA, USA). The survival rate was used to generate Kaplan-Meier survival curves which were compared using the log-rank (Mantel-Cox) test. Values are presented as the means (± SEM) and were analyzed using paired or unpaired t-tests or the corresponding Mann–Whitney U-test or a Wilcoxon–Mann–Whitney test. For multiple comparisons, we used Kruskal–Wallis testing. Survival curves comparing the effect of HSCT on survival of *Atm*-deficient mice were calculated by the Kaplan–Meier method. The following symbols indicate significant P-values: ∗*p <*0.05, ∗∗*p <*0.01 and ∗∗∗*p <*0.001.

## Results

### T-Cell Proliferation in the MLR

**Figure 1:**
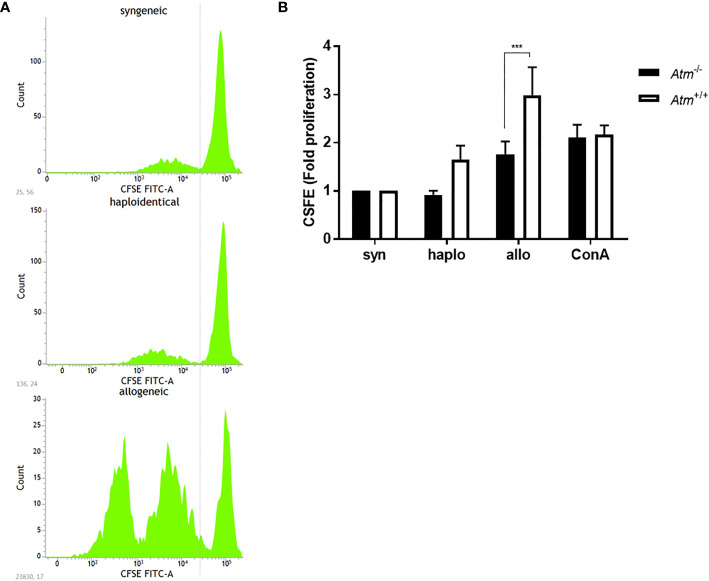
Proliferation of DCs in *Atm* ^-/-^ and *Atm* ^+/+^ mice following stimulation with syngeneic, haploidentical and allogeneic T-cells *in vitro*. **(A)** Representative CFSE intensity histograms showing the mean level of the CFSE expression per generation of T-cells isolated from spleens of syngeneic, haploidentical and allogeneic donor mice. **(B)** Cell proliferation of T-cells isolated from spleens of syngeneic, haploidentical and allogeneic donor mice and cocultured with DCs isolated from *Atm* ^-/-^ and *Atm* ^+/+^ mice (n = 8) was followed for 4 days using the CFSE cell proliferation kit. Data were normalized to the syngeneic T-cells and compared to haploidentical and allogeneic T-cell proliferative capacity. Data are presented as mean ± SEM. ***p < 0.001. CFSE, carboxy-fluorescein diacetate succinimidyl ester; DC, dendritic cell.

MLR was performed to test T-cell proliferation in a syngeneic, haploidentical and allogeneic setting *in vitro*. The MLR led to an elevated proliferative response of allogeneic donor T-cells compared to the proliferation of syngeneic and haploidentical donor cells (**Figure 1A**). The response of syngeneic compared to allogeneic donor T-cells was lower in dendritic cells derived from *Atm*-deficient mice compared to those from wild-type controls (*Atm* ^+/+^: 2.99 ± 0.58 fold proliferation, *Atm* ^-/-^: 1.76 ± 0.27 fold proliferation, *p* <0.001, **Figure 1B**).

### Survival, Body Weight and GvHD Status After HSCT

**Figure 2:**
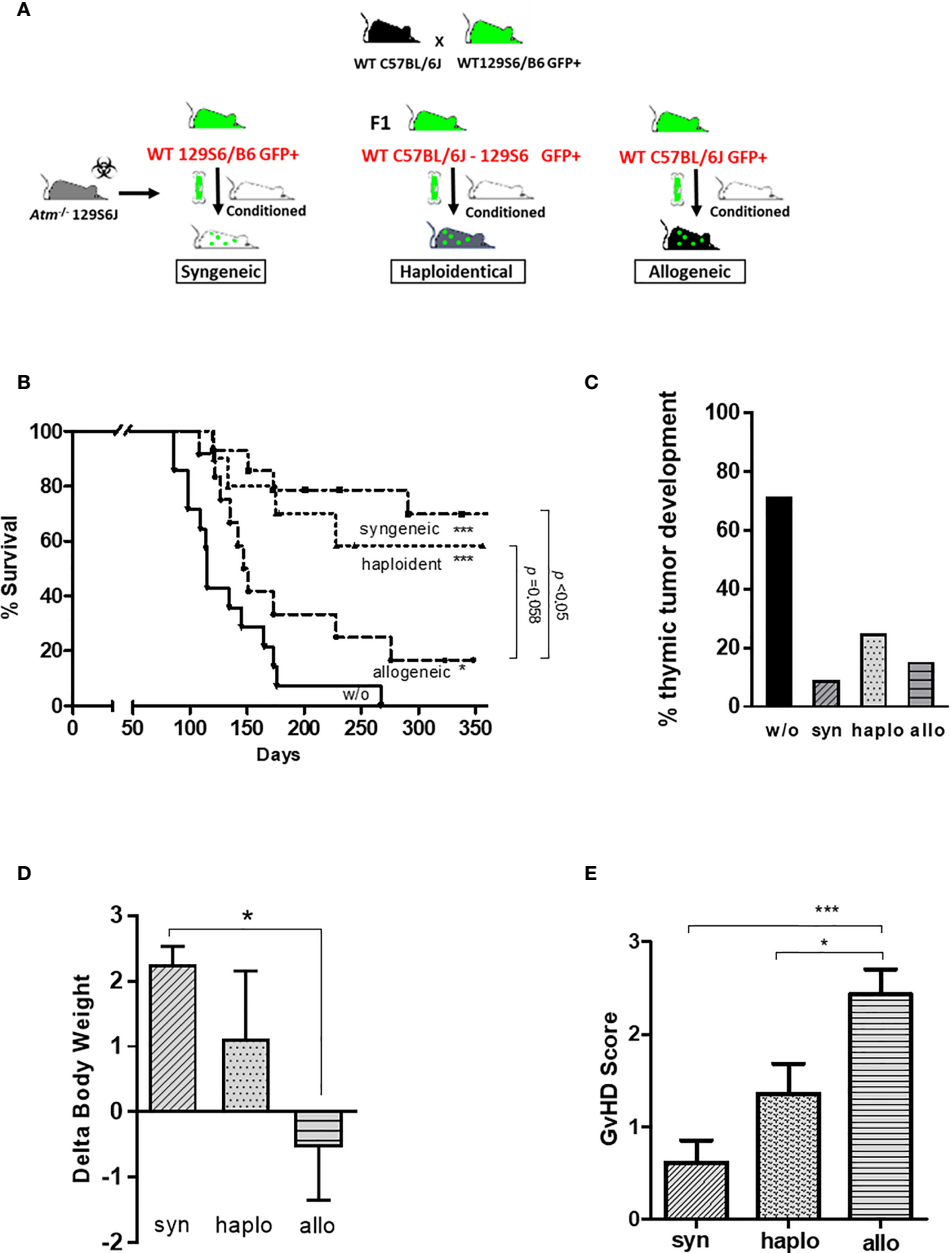
Syngeneic, haploidentical and allogeneic HSCT in *Atm*-deficient mice. **(A)** *Atm*-deficient recipients received a nonmyeloablative conditioning regimen consisting of anti-CD4 mAb and anti-CD8 mAb together with cyclophosphamide. Lin^-^ selected BMDCs derived from 129/SvEv GFP-transgenic WT mice (syngeneic setting) or derived from mice of the F1 generation of 129/SvEv GFP-transgenic and C57BL/6 WT mice (haploidentical setting), or from WT C57BL/6 mice GFP-transgenic (allogeneic setting) was used for HSCT. **(B)** Survival curves showed as Kaplan-Meier plots derived from *Atm* ^-/-^ mice (w/o; n = 14) before and after syngeneic (n = 11), haploidentical (n = 12) and allogeneic HSCT (n = 13) (* compared to w/o). **(C)** Percentage of thymic tumor development in untreated *Atm* ^-/-^ (w/o) and syngeneic, haploidentical or syngeneic transplanted *Atm* ^-/-^ mice. **(D)** Changes in body weight of *Atm* ^-/-^ mice receiving either BMDCs originated from syngeneic, haploidentical or allogeneic donor mice on the day of the transplantation compared to the end of experiments. **(E)** GvHD score grading performed throughout the observation period after syngeneic, haploidentical and allogeneic HSCT in *Atm* ^-/-^ mice. Data are presented as mean ± SEM. *p < 0.05, ***p < 0.001. BMDC, bone marrow-derived cells; GFP, green fluorescent protein; GvHD, Graft versus Host Disease; HSCT, hematopoietic stem cell transplantation; mAb, monoclonal antibody; WT, wild-type.

To address the different approaches of HSCT, *Atm*-deficient mice received hematopoietic BMDCs from syngeneic, haploidentical and allogeneic donor mice (**Figure 2A**). All transplantation settings significantly reduced the mortality rate and inhibited the tumor development in *Atm*-deficient compared to untreated *Atm*-deficient mice (**Figures 2B, C**). The survival rate was higher in the syngeneic (*p* <0.05) and haploidentical (*p* =0.058) HSCT compared to allogeneic HSCT.

Syngeneic and haploidentical HSCT improved the weight gain of *Atm*-deficient mice compared to the allogeneic transplantation setting in the indicated observation period (**Figure 2D**; syn: 2.26 ± 0.27 Δbody weight; haplo: 1.12 ± 1.04 Δbody weight; allo: -0.54 ± 0.81 Δbody weight, *p <*0.05). *Atm*-deficient mice receiving allogeneic BMDCs revealed a significant increased GvHD score than recipient mice receiving syngeneic and haploidentical BMDCs (syn: 0.62 ± 0.24 GvHD score, haplo: 1.36 ± 0.32 GvHD score, allo: 2.43 ± 0.27 GvHD score, *p* <0.05 and *p* <0.001; **Figure 2E**).

### T-Cells and T-Cell Chimerism

**Figure 3:**
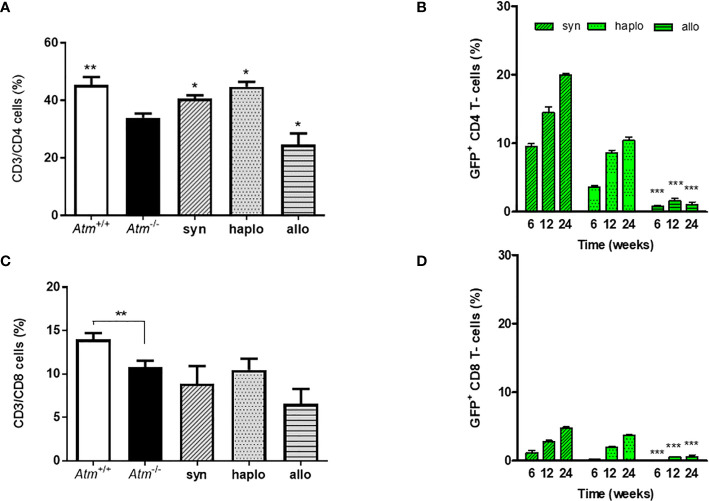
Phenotypic analysis of wild-type and *Atm*-deficient mice before HSCT and long-term engraftment of GFP expressing donor cells in *Atm*-deficient mice after syngeneic, haploidentical and allogeneic HSCT in peripheral blood samples. **(A, B)** Percentage of CD4^+^ and CD8^+^ lymphocytes in *Atm* ^-/-^ (n = 8) compared to *Atm* ^+/+^ mice (n = 8) as well as in syngeneic (n = 4), haploidentical (n = 4) and allogeneic (n = 4) transplanted *Atm* ^-/-^ 24 weeks post transplantation. **(C, D)** Analysis of the engraftment of GFP^+^ cells derived from syngeneic, haploidentical and allogeneic donor mice in peripheral blood samples of *Atm* ^-/-^ recipient mice (n = ≥ 5) and surface stained for CD4^+^ and CD8^+^ at timepoints 6, 12 and 24 weeks post HSCT (* only compared to syngeneic). Data are presented as mean ± SEM. *p < 0.05, **p < 0.01, ***p < 0.001. GFP, green fluorescent protein; HSCT, hematopoietic stem cell transplantation.

In the peripheral blood the percentage of CD3/CD4^+^ helper T-cells and CD3/CD8^+^ cytotoxic T−cells was significantly reduced in *Atm*-deficient mice prior to HSCT (CD4^+^: *Atm* ^+/+^ 45.34 ± 2.74%, *Atm* ^-/-^ 33.92 ± 1.46%; CD8^+^: *Atm* ^+/+^ 13.98 ± 0.73%, *Atm* ^-/-^ 10.78 ± 0.75%, *p <*0.01; **Figures 3A, B**). Syngeneic and haploidentical HSCT increased the percentage of CD4^+^ helper T-cells significantly compared to untreated *Atm*-deficient mice (CD3^+^/CD4^+^: *Atm* ^-/-^ 33.92 ± 1.46%, *Atm* ^-/-syn^ 40.58 ± 1.13%, *Atm* ^-/-haplo^ 41.22 ± 3.73%, *p* <0.05; **Figure 3A**). Interestingly, the percentage if CD4^+^ helper cells was significantly diminished compared to untreated *Atm*-deficient mice (CD3^+^/CD4^+^: *Atm* ^-/-^ 33.92 ± 1.46%, *Atm* ^-/-allo^ 24.61 ± 3.88%, *p* <0.05; **Figure 3A**). Tracking of GFP^+^ cells revealed an increase in the CD4^+^ and CD8^+^ subpopulation 12 months after syngeneic and haploidentical HSCT. In contrast, the percentage of GFP^+^ T-cells was significantly lower after allogeneic HCST at all measured time points and showed no increase over time (**Figures 3C, D**).

### T-cell Activation of ATM-Competent Cells in Transplanted *Atm*-Deficient Mice

**Figure 4:**
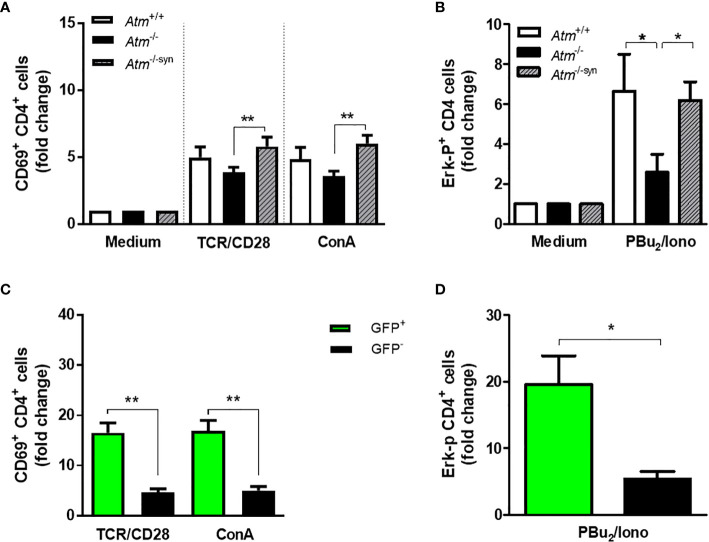
TCR-dependent T-cell activation and Expression of the phosphorylated (p)-Erk protein in *Atm*-deficient mice after syngeneic HSCT. **(A)** Flow cytometric analysis of T-cell activation in CD4^+^ splenocytes from *Atm* ^+/+^ (n = 7), *Atm* ^-/-^ (n = 7) and syngeneic transplanted *Atm* ^-/-^ mice (n = 9, *Atm* ^-/-syn^) 12 weeks post HSCT using CD69 as activation marker. The cells were stimulated either *via* TCR/CD28 or *via* ConA for 4 h *in vitro* and the data were normalized to the medium control. **(B)** Expression of the phosphorylated (p)-Erk protein in stimulated CD4^+^ splenocytes from *Atm* ^+/+^ (n = 10), *Atm* ^-/-^ (n = 8) and *Atm* ^-/-syn^ (n = 9) 12 weeks after HSCT measured by flow cytometry. The cells were either stimulated *via* TCR/CD28 for 5 min and PBu_2_/Iono for 15 min *in vitro*. The data were normalized to the unstimulated medium control. **(C)** T-cell activation in GFP^+^ and GFP^-^ CD4^+^ T-cells from *Atm* ^-/-syn^ mice stimulated either directly *via* αCD3 and αCD28 or nonspecifically by ConA for 4 h 12 weeks after HSCT. **(D)** Expression of the signaling protein Erk in GFP^+^ and GFP^-^ CD4^+^ T-cells from *Atm* ^-/-syn^ stimulated *via* PBu_2_/Iono. Data are presented as mean ± SEM. *p < 0.05, **p < 0.01. ConA, Concanavalin A; GFP, green fluorescent protein; HSCT, hematopoietic stem cell transplantation; TCR, T cell receptor.

CD4 T-cell activation was evaluated by CD69 expression and activation of the MAPK/ERK pathway in isolated splenocytes derived from syngeneic transplanted *Atm-*deficient mice and compared to those from either wild-type or *Atm*-deficient mice. Activation *via* TCR/CD28 (*Atm* ^-/-^: 3.87 ± 0.39 fold change, *Atm* ^-/-^trans: 5.8 ± 0.7 fold change, *p <*0.01) as well as through the TCR cross-linker ConA (*Atm* ^-/-^: 3.61 ± 0.36 fold change, *Atm* ^-/-syn^: 6.03 ± 0.61 fold change, *p <*0.01) resulted in a significant increase in CD69 expression compared to the CD69 expression in untransplanted *Atm-*deficient mice (**Figure 4A**).

CD69 expression was significantly higher in ATM-competent (GFP^+^) splenocytes than in *Atm*-deficient cells (GFP^-^) in TCR-stimulated (GFP^-^: 4.69 ± 0.71 fold change, GFP^+^: 16.54 ± 1.99 fold change, *p <*0.01) and ConA-stimulated cells (GFP^-^: 5.05 ± 0.82 fold change, GFP^+^: 16.95 ± 2.07 fold change, *p <*0.01; **Figure 4C**).

HSCT rescued the ERK-phosphorylation signal of CD4^+^ splenocytes in *Atm*-deficient mice (**Figure 4B**). The stimulation of CD4^+^ T-cells using PBu_2_/Ionomycin resulted in a 3.61-fold higher phosphorylation of Erk in transplanted *Atm*-deficient mice 12 weeks after HSCT compared to cells from untreated *Atm*-deficient mice (*Atm* ^-/-^: 2.59 ± 0.9 fold change; *Atm* ^-/-syn^: 6.2 ± 0.9 fold change; *p* <0.05; **Figure 4B**). The GFP^+^ CD4 T-cell population showed a significantly increased Erk-phosphorylation signal compared to the GFP^-^ CD4^+^ T-cells (GFP ^-^: 5.38 ± 1.16 fold change; GFP^+^:19.6 ± 4.32 fold change; *p *<0.05; **Figure 4D**).

### Activation of Naïve and Memory CD4 T-Cells From Transplanted *Atm*-Deficient Mice

**Figure 5:**
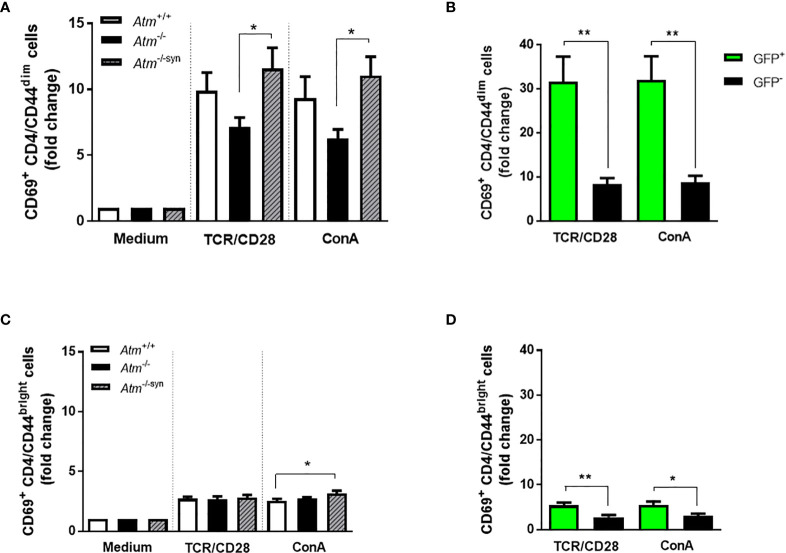
Activation of T-cell subpopulations in splenocytes of *Atm*-deficient mice *in vitro* using flow cytometry. **(A)** Expression of CD69^+^ in TCR- and ConA-stimulated CD4^+^/CD44^dim^ splenocytes from untreated *Atm* ^+/+^, *Atm* ^-/-^ and transplanted *Atm* ^-/-syn^ mice 12 weeks after HSCT (n > 6). The data were normalized to the unstimulated medium control. **(B)** Expression of CD69^+^ in GFP^+^ and GFP^-^ CD4^+^/CD44^dim^ splenocytes stimulated either *via* TCR or ConA isolated from *Atm* ^-/-syn^ mice 12 weeks after HSCT (n = 6). **(C)** T-cell activation in CD4^+^/CD44^bright^ splenocytes stimulated with either αCD3 and αCD28 or ConA from *Atm* ^+/+^, *Atm* ^-/-^, and *Atm* ^-/-syn^ mice (12 weeks after HSCT) (n > 6). The data were normalized to the medium control. **(D)** Flow cytometric analysis of CD69^+^ expression GFP^+^ and GFP^-^ CD4^+^/CD44^bright^ splenocytes isolated from *Atm* ^-/-syn^ mice either stimulated *via* TCR or ConA 12 weeks after HSCT (n = 6). Data are presented as mean ± SEM. *p < 0.05, **p < 0.01. ConA, Concanavalin A; GFP, green fluorescent protein; HSCT, hematopoietic stem cell transplantation; TCR, T cell receptor.

As previously described by our group, HSCT increases naïve CD4^+^/CD44^dim^ T-cells rather than memory CD4^+^/CD44^bright^ cells in *Atm*-deficient mice (14). To examine whether this fact is also visible on CD4 T-cell activation, CD4^+^/CD44^dim^ and CD4^+^/CD44^bright^ T-cell subpopulations derived from the spleen of *Atm*-deficient mice, wild-type mice and syngeneic transplanted *Atm*-deficient mice were either activated *via* TCR or with ConA evaluating CD69 expression using flow cytometry. Syngeneic HSCT significantly increased CD69 expression on CD4^+^/CD44^dim^ (TCR/CD28: *Atm* ^-/-^: 7.14 ± 0.71 fold change, *Atm* ^-/-syn^: 11.59 ± 1.57 fold change, *p <*0.05; ConA: *Atm* ^-/-^: 6.26 ± 0.70 fold change, *Atm* ^-/-syn^: 11.05 ± 1.42 fold change, *p <*0.05), but only to a small extent on CD4^+^/CD44^bright^ T cells (**Figures 5A, C**).

In both subpopulations, CD69 activation was significantly increased in GFP^+^ cells from *Atm−*deficient mice after syngeneic HSCT compared to the GFP^-^ cell fraction after stimulation *via* TCR/CD28 (CD4^+^/CD44^dim^ cells; GFP^-^: 8.35 ± 1.41 fold change, GFP^+^: 31.60 ± 5.69 fold change, *p* <0.01; CD4/CD44^bright^ cells; GFP^-^: 2.79 ± 0.46 fold change, GFP^+^: 5.41 ± 0.66 fold change, *p* <0.01) and after ConA stimulation (CD4/CD44^dim^ cells; GFP^-^: 8.73 ± 1.55 fold change, GFP^+^: 31.99 ± 5.36 fold change, *p* <0.01; CD4/CD44^bright^ cells; GFP^-^: 3.01 ± 0.53 fold change, GFP^+^: 5.52 ± 0.76 fold change, *p <*0.05; **Figures 5B, D**).

### Heterozygous Syngeneic HSCT in *Atm*-Deficient Mice

**Figure 6:**
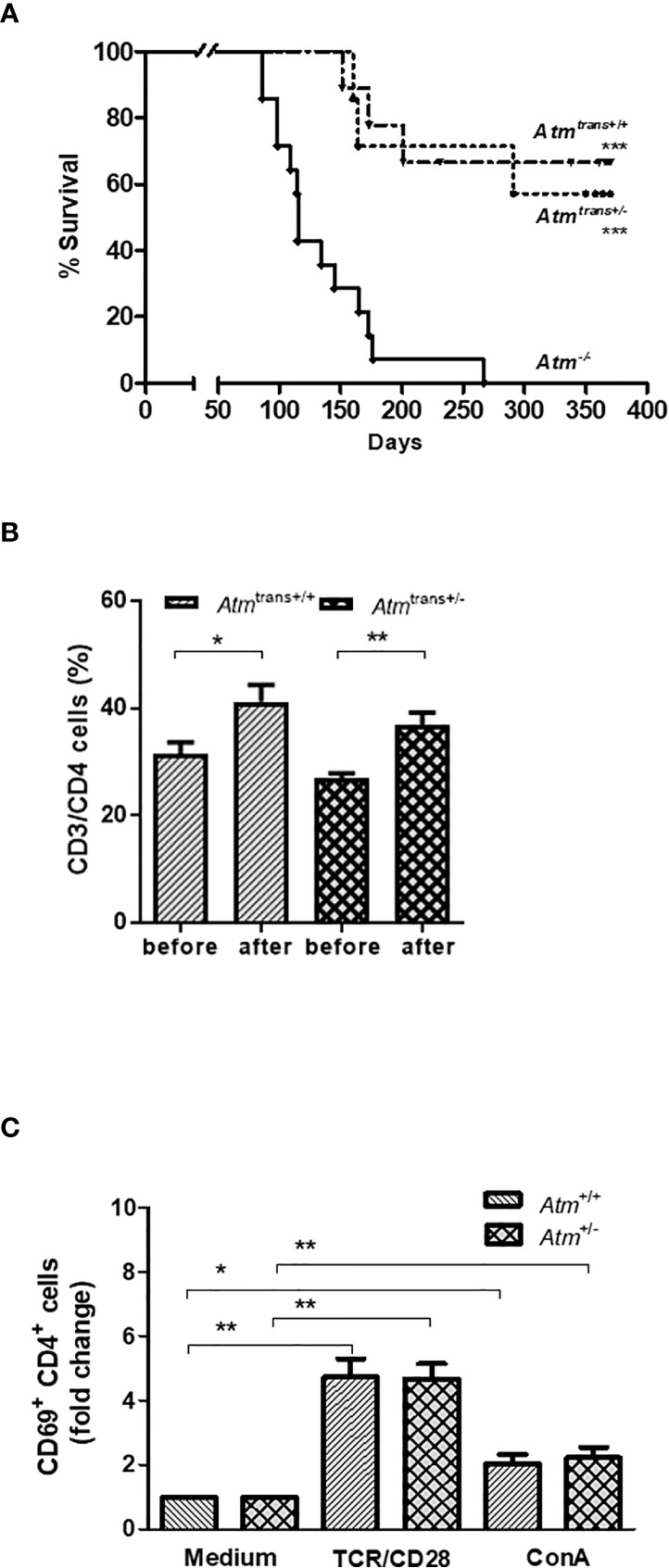
Effects of HSCT with BMDCs isolated from heterozygous (*Atm* ^+/-^) donors on survival and chimerism in *Atm*-deficient mice and functional T-cell activation of splenocytes from *Atm* ^+/-^ mice *in vitro*. **(A)** Survival curves shown as Kaplan-Meier plot from *Atm* ^-/-^ mice after heterozygous (*Atm* ^+/-^, n = 7) or homozygous (*Atm* ^+/+^, n = 9) HSCT and *Atm* ^-/-^ without treatment (n = 14). **(B)** Phenotypic analysis of CD4^+^ T cells in peripheral blood taken from *Atm*-deficient mice before and 24 weeks after receiving BMDCs from *Atm* ^+/-^ or *Atm* ^+/+^ donors (n ≥ 4) using flow cytometry. **(C)** Functional analysis of CD4^+^, CD4^+^/CD44^dim^ (naïve) and CD4^+^/CD44^bright^ (memory) CD69 T-cell activation in splenocytes isolated from *Atm* ^+/-^ and *Atm* ^+/+^ mice stimulated with either TCR/CD28 or ConA for 4 h *in vitro* (n = 5). The data were normalized to the medium control. Data are presented as mean ± SEM. *p < 0.05, **p < 0.01. ConA, Concanavalin A; GFP, green fluorescent protein; HSCT, hematopoietic stem cell transplantation; TCR, T cell receptor.

This study also examined the effects of grafts from asymptomatic heterozygous donors (*Atm* ^+/-^) on survival, immunophenotyping and T lymphocyte activation in *Atm*-deficient mice. Interestingly, the syngeneic HSCT consisting of donors heterozygous for *Atm* significantly prolonged the lifespan of *Atm*-deficient mice as well as HSCT with homozygous *Atm* ^+/+^ donors (**Figure 6A**). In addition, the HSCT using *Atm* ^+/-^ donors restored the CD4^+^ T-cell population significantly in *Atm*-deficient mice 24 weeks after HSCT compared to the T-cell status before HSCT (*Atm* ^+/-^ before: 27.03 ± 0.81%, after *Atm* ^+/-^: 36.81 ± 2.34%; *p <*0.01; **Figure 6B**). The functional analysis of the CD4^+^ T-cell activation after either direct stimulation *via* TCR/CD28 or ConA *in vitro* revealed no differences between splenocytes isolated from heterozygous *Atm*-mice compared to homozygous *Atm*-mice (**Figure 6C**).

## Discussion

A-T with its multiple genetic defects results in disruption of DNA repair processes leading to T−cell lymphopenia and impaired antibody production due to a V(D)J recombination defect during lymphocyte maturation (2, 22). This recombination defect is responsible for the immunodeficiency and the high risk of cancer in the immune cell compartment (23). As currently no curative therapy for A-T exists, new treatment strategies are necessary. HSCT, as it is carried out in other genomic instability syndromes as Fanconi anemia or Nijmegen Breakage Syndrome, could be a promising approach, although it is controversially discussed as the survival rate in patients after transplantation is only 25% (24–27). Conditioning is a critical factor in the clinical setting of SCT in A-T. The hypersensitivity to ionizing radiation and radiometric drugs results in a high risk of mortality. Therefore, the selection of a non-myeloablative condition is inevitable before considering SCT in A-T patients. In this regard it is worth to note that potential neurotoxic effects of busulfan-based protocols in patients with underlying neurodegenerative conditions must viewed with caution and should be generally avoided (15, 28, 29). In these patients, the suitability for SCT is controversial and the benefits of SCT are difficult to verify in case reports. The indication should be made cautiously and considered in each individual case. Nevertheless, SCT may be beneficial to improve immunodeficiency and control the risk of lymphoid malignancies and basic research may have the potential to identify some patients as beneficiaries of SCT.

Regarding patient selection, A-T patients with recurrent infections, severe manifestations of immunodeficiency and children with a high risk of lymphoid malignancies are the most likely candidates to benefit from SCT therapy, based on reported data.

According to the literature, HSCT in A-T is not able to ameliorate neurological symptoms, but researchers state cautiously optimistic that the patients exhibit a milder progression of ataxic symptoms and slowed neurologic deterioration (15, 28, 29).

Allogeneic HSCT often results in a severe GvHD, which is the leading complication and cause of non-relapse mortality in the patients (17). Recent clinical progress has been made in the use of transplants from partially matched or haploidentical related donors for patients who are in need of a stem cell transplant but do not have a HLA-matched related or unrelated donor (17, 30). Thus, over the last recent years the use of haploidentical donors has increased as alternative of allo-HSCT since, advantageously haploidentical donors are available for 95% of the patients (17, 31–33). In A-T, haploidentical HSCT has been successfully performed in two patients in Frankfurt who received allo-HSCT from a matched sibling donor (14, 15). However, experience in the transplantation strategy for A-T patients is still scarce (29).

The aim of the present study was to investigate the effects of different HSCT strategies on survival and functional reconstitution of the immune system in *Atm*-deficient mice. In recent years, we and others were able to show that HSCT significantly rescues the T-cell department and prevents the development of thymic tumors in *Atm*-deficient recipient mice (13, 14, 19, 20). However, all experiments in *Atm*-deficient mice were performed in a syngeneic setting. In this study, we compared haploidentical and allogeneic HSCT to the syngeneic setting in these mice. Usually, *Atm*-deficient mice die from thymic lymphomas at the age of 3-6 months, but however, bone marrow transplantation with ATM-competent cells leads to a stable chimerism and prevents tumorigenesis and prolongs the lifespan of the animals significantly (20). As one might expect, our present study demonstrated that allo-HSCT was associated with an increased death rate compared to the syngeneic setting. In fact, allogeneic transplanted mice showed a reduction in tumor expression, but the mice exhibited significant weight loss (>20%), showed a higher incidence of skin alterations compared to their syngeneic or haploidentical transplanted littermates and exhibited an impaired hematopoiesis due to graft failure (34, 35).

Further, our *in vitro* MLR experiments revealed that allogeneic-derived ATM-competent T−cells show a lower immunological proliferative activity on *Atm*-deficient DCs compared to wild-type DCs assuming a low defensive reaction. In contrast, like syngeneic HSCT, haploidentical HSCT significantly extended the lifespan of *Atm*-deficient mice through the reduction of thymic tumors. Inhibited tumorigenesis and prolonged survival of the *Atm*-deficient mice in the haploidentical setting is in line with the finding that haploidentical HSCT is a valuable alternative for patients with hematological disorders lacking a HLA-matched unrelated donor (36, 37). Survival was accompanied by an engraftment of donor CD4^+^ lymphocytes at the level which was reached after syngeneic HSCT (20, 38). Our data show that haploidentical HSCT can fill the gap of the extreme cell loss in the naïve T-cell populations. In addition to the reduction in the numbers of naïve (CD45RA) CD4^+^ as well as CD8^+^ T-cell subsets, an intrinsic deficiency in the signal transduction pathway proximal to the PKC has been described in peripheral blood mononuclear cells (PBMCs) from patients with A-T (2). Cellular activation indicated by CD69 expression was reduced compared to controls suggesting an early activation defect in A-T lymphocytes (2). Our analysis provided evidence that syngeneic HSCT can increase T-cell activation and signal transduction to the level of wild-type mice and can rescue the functionality of the T-cell compartment in *Atm*-deficient mice. Improvement of the T-cell activity was predominantly found in the naïve CD4 cell population (14). However, it must be noted, that the present study did not evaluate cellular and functional T-cell activation in *Atm*-deficient mice after haplo-HSCT representing a potential weakness of the study design. In addition, beside *ex vivo* experiments, only *in vivo* studies can ultimately show the restored functionality of the immune system after transplantation, e.g., by using a lymphocytic choriomeningitis virus (LCMV) transfected murine lung carcinoma cell tumor (39).

Although, outcomes of haploidentical HSCT have improved with remarkable progress within the last years, and HLA-haploidentical donors such as biological parents, biological children, full or half siblings, or collateral related donors might be an acceptable source for donor cells, in A-T, haploidentical donors have a very high probability of heterozygosity for *ATM* (40). Indeed, since haploidentical donors are carriers of only one ATM allele the outcome of HSCT has not been examined so far. Phenotypically, *Atm-*heterozygotes mice do not demonstrate reduction in size and body weight compared to wild-type counterparts (41). Cells from heterozygous and wild-type mice showed similar patterns of cell surface marker expression and chromosomal instability (19, 41). Our transplantation experiments let to a significantly improved survival of *Atm*-deficient mice transplanted with cells from a heterozygous donor equivalent to transplantation with wild-type donor cells. Both transplantation settings significantly increased CD4 cell numbers as well as CD4 cell functionality without any differences between the genotype of the donor cells. However, it is a well-known fact that the results found in murine animal models cannot directly be applied to humans and even that our *Atm*-deficient mouse model recapitulates the A-T phenotype, only short-term rather than long-term complications after HSCT can be studied. ATM-heterozygous alterations are postulated to be predispose to epithelial neoplasia, in particular breast cancer and a higher rate of lymphoid malignancies are also in the focus of discussion (42). Loss of heterozygosity happens frequently in sporadic tumors of lymphoid origin, and a high prevalence of ATM gene alterations in diverse sporadic lymphoproliferative disorders has been reported (23). In this regard, screening of ATM heterozygosity in potential donors for HSCT in A-T patients has to be considered (42). Our study demonstrates that haploidentical HSCT is a feasible strategy for A-T, even if the donor is heterozygous for ATM regarding restoration of lymphocyte populations and improvement of T-cell functionality. The project delivers substantial data about feasibility, safety, and efficacy of allogeneic and haploidentical HSCT in A-T. However, this basic research cannot substitute any research in humans and further studies are needed to develop a prospective study protocol for HSCT in children with A-T.

## Data Availability Statement

The datasets generated for this study are available on request to the corresponding author.

## Ethics Statement

This animal study was reviewed and approved by the German Animal Subjects Committee (Gen. Nr. FK/1034).

## Author Contributions

RD and RS conceptualized and designed the study and wrote the manuscript. RD, LG, and PB helped with acquisition of data. RD, LG, PB, RS, and SZ analyzed and interpreted the results. RD, RS, and SZ did the conceptualization, the methodology, and the writing-review and editing of the manuscript. RD, LG, and PB helped with acquisition and validation, formal analysis,visualization, and data curation. RD and RS wrote the original draft. RS and SZ did the supervision and the project administration. RS acquired the funding for the project. All authors contributed to the article and approved the submitted version.

## Funding

This study was funded by the SPARKS Charity, Action for A-T (Grant Reference 14GOU01, London, England).

## Conflict of Interest

The authors declare that the research was conducted in the absence of any commercial or financial relationships that could be construed as a potential conflict of interest.
